# Study on metabonomics of Chinese herbal medicine in the treatment of type 2 diabetes mellitus complicated with community-acquired pneumonia

**DOI:** 10.1097/MD.0000000000022160

**Published:** 2020-09-11

**Authors:** Hongjing Yang, Rensong Yue, Jie Zhou, Zhu Zeng, Lizhen Wang, Xiaoqin Long, Ning Ding, Xiaoying Huang

**Affiliations:** a Hospital of Chengdu University of Traditional Chinese Medicine; b Chengdu Qingbaijiang District Traditional Chinese Medicine Hospital, Chengdu, China.

**Keywords:** Chinese herbal medicine, community-acquired pneumonia, randomized controlled trial, type 2 diabetes mellitus

## Abstract

**Introduction:**

Community-acquired pneumonia (CAP) is the main acute complication of type 2 diabetes mellitus (T2DM) and the main cause of hospitalization for infectious diseases. Unfortunately, in the treatment of type 2 diabetes mellitus complicated with community-acquired pneumonia (T2DM-CAP), modern medicine is still faced with enormous challenges because of insulin resistance and drug-resistant bacteria. In recent decades, clinical and experimental evidence shows that Chinese herbal medicine (CHM) has a certain beneficial effect on diabetes and pneumonia. Therefore, this trial aims to assess the efficacy and safety of CHM plus western medicines for the treatment of T2DM-CAP.

**Methods:**

We propose a double-blind, placebo-controlled, randomized superiority trial.

A total of 92 participants with T2DM-CAP will be randomly allocated at a 1:1 ratio to either the experimental group, which will receive modified Ban-Xia-Xie-Xin-Decotion and basic treatment, or the control group, which will receive basic treatment only. The study duration will be 14 days. The primary outcome will be the total clinical effective rate. The secondary outcomes are traditional Chinese medicine symptom score scale, pneumonia severity index, usage time of antibiotic, time required for blood sugar to reach the required level, frequency of hypoglycemia, and chest CT. Liquid chromatograph-mass spectrometry method will be used to explore the blood metabolism profiles of the subjects, to explore the pathogenesis of T2DM-CAP and the mechanism of CHM on T2DM-CAP. Adverse events will also be evaluated.

**Discussion:**

This trial will provide evidence of the effectiveness and safety of traditional CHM in treating patients with T2DM-CAP.

**Trial registration number:**

ChiCTR2000035204

## 1 Introduction

With about half a billion people affected, type 2 diabetes mellitus (T2DM) has become the leading chronic and non-communicable disease worldwide and been associated with high morbidity and mortality.^[1–2]^ In China, the prevalence of type 2 diabetes is as high as 10.4%, with more than 100 million people, accounting for about a quarter of the global total, and this number is expected to maintain an upward trend.^[1–2]^ Metabolic disorders caused by diabetes give rise to multiple organ injury. In addition, several aspects of immunity are changed in patients with diabetes. For instance, the normal role of polymorphonuclear leukocytes, leukocyte chemotaxis, adherence, and the processes of phagocytosis may be compromised.^[3–6]^ Antioxidant systems involved in bactericidal activity may also be damaged.^[7]^ The impact of these disorders on the development and prognosis of diabetes is immeasurable.

With the increase of the prevalence of diabetes, the incidence of community-acquired pneumonia (CAP) also increases significantly.^[8–9]^ CAP is a primary infectious cause of hospitalization worldwide.^[10–12]^ Previous studies have manifested that diabetes is a major risk factor for pneumonia-related hospitalizations.^[13–14]^ Individuals with DM may increase their susceptibility to pulmonary infection due to various factors, including risk of aspiration, impaired lung function, dysfunctional immunity related to the harmful effects of hyperglycaemia, and other co-existing comorbidities.^[14–15]^ Multiple studies have demonstrated that diabetics have a longer hospital stays, a higher risk of death from CAP,^[16–18]^ and a higher healthcare costs than non-diabetic patients.^[19–20]^

At present, modern medical treatment of T2DM-CAP is mainly symptomatic treatment, that is, timely use of antibiotics to control infection on the basis of controlling the level of blood glucose.^[21–22]^ Although comprehensive measures can achieve a certain effectiveness, there are still many difficulties in the treatment of the disease due to the emergence of insulin resistance and drug-resistant bacteria.^[11,23]^ Such as the repeated use of broad-spectrum antibiotics can easily induce flora imbalance and mutation strains. What is more, even if blood sugar and inflammation are controlled, there is still no significant improvement in the patient's condition. There is an urgent need for new treatment plans to improve the overall control and prognosis of T2DM-CAP and reduce medical costs.

Considering the risk of antibiotic resistance and insulin resistance, the combined use of drugs is gradually becoming more popular. Chinese herbal medicine (CHM) has been used for thousands of years for treating pneumonia or diabetes. In recent decades, evidence from both clinicians and patients suggests that CHM has some beneficial effect on CAP^[24–25]^ and diabetes.^[26–27]^ Our research group has been engaged in diabetes research for nearly 20 years. Ban-Xia-Xie-Xin-Decotion (BXXXD) is composed of 7 herbs and has been proved to have good treatment effects both in clinic and experimental studies.^[28–30]^ Modern pharmacological studies have shown that the formula has multiple effects such as anti-inflammatory, antioxidation, antitussive, and immunomodulatory.^[31–33]^ Nevertheless, the effects of modified BXXXD on the clinical prognosis of CAP patients with diabetes remain unclear. Thus, this trial aims to assess the efficacy and safety of modified BXXXD plus western medicines for the treatment of T2DM-CAP. In addition, Blood metabolism profiles of participants by liquid chromatograph-mass spectrometer method will be measured to explore the pathogenesis of T2DM-CAP and the mechanism of drug action.

## 2 Methods/design

### 2.1 Design

**Figure 1:**
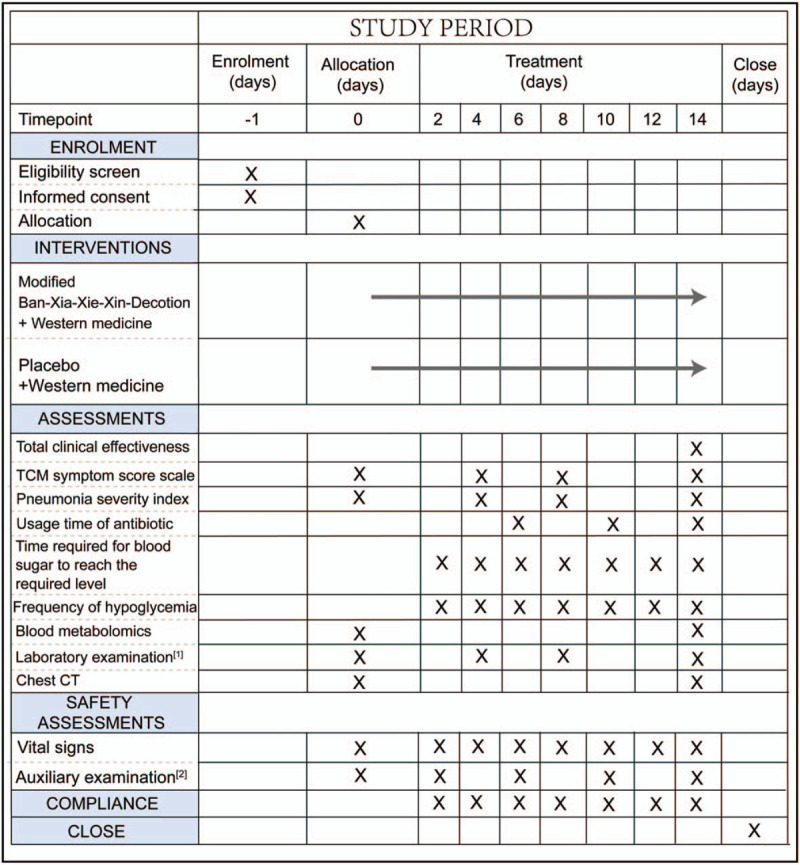
Spirit figure of enrollment, interventions, and assessments.^[1]^ Laboratory examination: including white blood cell count, percentage of neutrophils, C-reactive protein and procalcitonin.^[2]^ Auxiliary examination: blood, urine, feces, electrocardiogram, kidney, and liver function.

**Figure 2:**
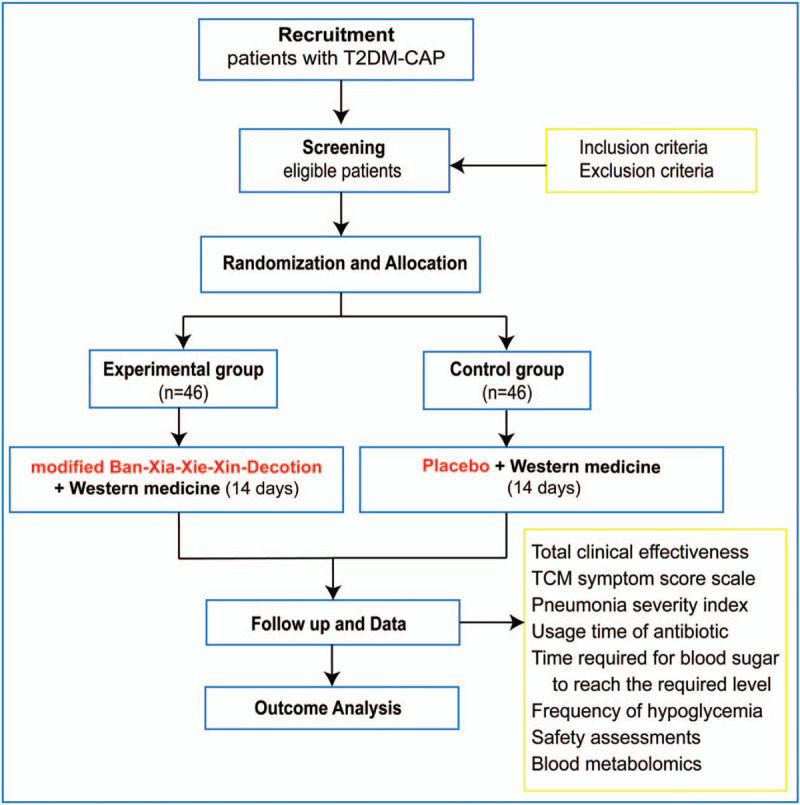
Flowchart of the study design. CAP = community-acquired pneumonia, T2DM = type 2 diabetes mellitus, TCM = traditional Chinese medicine.

This study is a randomized, double-blind, placebo-controlled clinical trial. This trial has been registered with the Chinese Clinical Trial Registry. After obtaining written informed consent, 92 eligible participants will be randomly assigned to the experimental group or control group in a 1:1 ratio. The 2 groups will then undergo a 2-week treatment period. The trial aims to investigate the additional benefits and safety of CHM plus western medicines compared with western medicine treatment only of T2DM-CAP, and to explore the mechanism in light of Metabonomics. The study will comply with the Standard Protocol Items: Recommendations for Interventional Trials 2013 statement (see Fig. 1 for the Standard Protocol Items: Recommendations for Interventional Trials figure of enrollment, interventions, and assessments). The research flow chart is illustrated in Figure 2.

### 2.2 Ethics approval

The final amendments (version: July 31, 2020) and the consent form have been reviewed and approved by the China Ethics Committee of Registering Clinical Trials (Approval No. ChiMCTR2000003540). The study will abide by the Declaration of Helsinki (Edinburgh 2000 version).

### 2.3 Recruitment

All patients with T2DM-CAP at endocrinology department and respiratory department in hospital of Chengdu University of Traditional Chinese Medicine (TCM) (Chengdu, China) will be screened and enrolled. Prior to enrollment, subjects will be informed about the detailed explanation of the clinical study, including the purpose, scheduling, and possible benefits and risks. All eligible participants will be required to sign an informed written consent before the start of the study process. Patient recruitment starts in August 2020 and is planned to be completed in August 2021.

### 2.4 Sample size

Sample size calculations are based on the primary outcome (the clinical effective rate at 2 weeks). According to the previous literature,^[34]^ we assumed the clinical effective rate of 73% in the control group and 93% in the experimental group over 14 days. Thus, a sample size of 82 patients is needed to achieve a statistical power of 80% (single-sided type-1 error of 5% in both scenarios). Assuming that 10% of patients are likely to be lost during follow-up, a total of 92 patients will be enrolled.

### 2.5 Randomization and allocation concealment

A member of Sichuan evidence-based Medicine Center of TCM, who does not participate in subsequent trials, uses SAS 9.2 software (SAS Institute Inc., Cary, NC) to generate 92 random serial numbers. Randomization is performed after screening and baseline evaluation, and eligible T2DM-CAP subjects will be randomized in a 1:1 fashion to receive 1 of 2 treatments. The group numbers are provided in continuously numbered, sealed envelopes made of carbonless paper. The envelopes will be held by a research administrator who will not be directly involved in the recruitment or follow-up of any participants. The administrator provides the subjects with their group number on the day of inclusion. Consequently, the subjects, clinical researchers, data managers, outcome assessors, and statisticians do not know the allocations, which not be revealed until the study is completed.

### 2.6 Blinding

This trial is a double-blind design and participants and statisticians will be blinded during the trial period. Both placebo granules and Chinese herbal granules are produced, packaged, and labeled by the same manufacturer to ensure identity in specification and appearance. In addition, the research team will be trained not to interflow with the participants about their probable treatment group allocation. Only an emergency occurs, such as serious adverse event, can the investigator inform the main investigator to decide whether to expose the blind.

### 2.7 Diagnostic criteria

Participants must meet the Western medicine diagnostic criteria for type 2 diabetes (Table 1),^[21]^ CAP (Table 2)^[22]^ and the TCM syndrome diagnostic criteria of pulmonary-splenic asthenia and accumulation of phlegm-dampness syndrome (Table 3).^[35]^ The determination of syndrome differentiation are determined independently by 2 appointed deputy physicians of TCM.

**Table 1:**
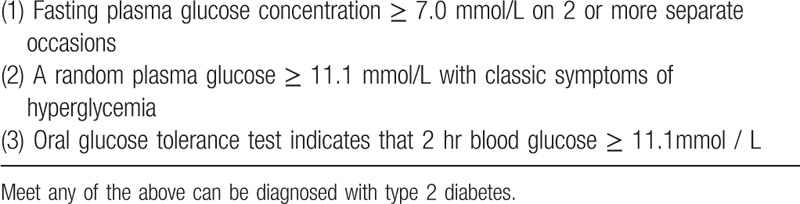
Diagnostic criteria for type 2 diabetes mellitus^[21]^.

**Table 2:**
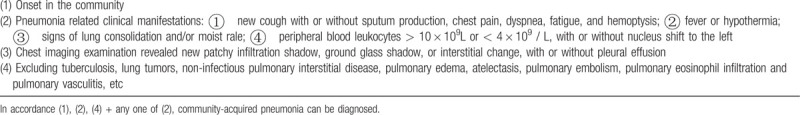
Diagnostic criteria for community-acquired pneumonia ^[22]^.

**Table 3:**
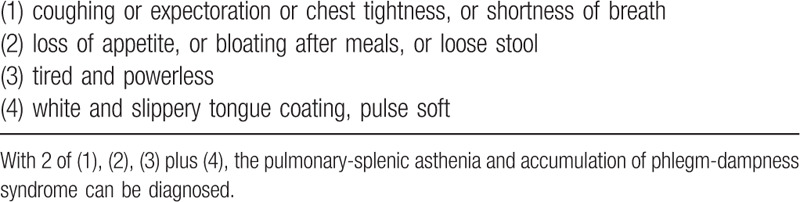
Diagnostic criteria for traditional Chinese medicine differentiation of pulmonary-splenic asthenia and accumulation of phlegm-dampness syndrome^[35]^.

### 2.8 Eligibility criteria

#### 2.8.1 Inclusion criteria

- Participants have a history of type 2 diabetes and are hospitalized with confirmed CAP.
- Participants meet the criteria for pulmonary-splenic asthenia and accumulation of phlegm-dampness syndrome in TCM.
- Participants aged 18 to 70 years, no gender limitations.
- Participants can complete this study and all tests.
- Participants provide informed written consent and volunteer to participate in the trial.

### 2.9 Exclusion criteria

- Patients with other coexistent pulmonary diseases (e.g., bronchiectasis, pulmonary edema, chronic obstructive pulmonary disease, tuberculosis, or interstitial lung disease).
- Patients with severe comorbidities, such as malignant tumors, infectious diseases, coronary heart disease, mental abnormality, or patients whose laboratory data indicating severe systemic disease (such as liver and kidney dysfunction).
- Women who are breastfeeding, or pregnant.
- Allergies for experimental drugs or CHM.
- Patients have participated in other clinical trials in the 1 month preceding the trial.

### 2.10 Termination and withdrawal criteria

All participants are informed of their right to terminate and withdraw from the trial. The reason will be recorded in case report forms (CRFs). The criteria for stopping treatment and withdrawing from the research are:

- participants suffered severe adverse reactions related to the medication, and the researcher believes that they should stop taking drugs;
- participants suffered from another serious illness that required treatment during the study;
- participants’ condition deteriorate severely and even require respiratory support therapy;
- poor compliance, the actual dose is less than 80% of the prescribed dose.

### 2.11 Test drugs

Test drugs are modified BXXXD and BXXXD mimetic agent (placebo), provided by the Sichuan Green Pharmaceutical Technology Development Co., Ltd. (Sichuan, China). Whole ingredients of modified BXXXD are Rhizoma Pinelliae Praeparata (Fa ban xia) 15 g, Zingiberis Rhizom (Gan Jiang) 10 g, Scutellariae Radix (Huang qin) 15 g, Coptidis Rhizoma (Huang lian) 6 g, Ginseng Radix et Rhizoma (Ren shen) 15 g, Jujubae Fructus (Da zao) 10 g, Radix Et Rhizoma (Zhi gan cao) 10 g, Ephedrae Herba (Ma huang) 15 g, Armeniacae Semen Amarum (Ku xing ren) 15 g, Poria (Fu Ling) 15 g, and Citri Reticulatae Percarpium (Chen pi) 15 g. The pharmaceutical company mixes, cooks, and sprays the herbs medicines in the prescription to form granules. These granules are packaged into small single-dose sachets, each weighing 10 grams. The placebo is made from starch with no active ingredients. By adding a diversity of food pigments, the placebo is as close to the real particles in appearance and taste as possible.

## 3 Interventions

### 3.1 Treatment plan

Both groups are given basic treatment^[21–22]^: on the basis of lifestyle interventions (diabetes diet, proper exercise), short-acting insulin subcutaneous injection is preferred to ensure that blood glucose can be controlled within the target range (FPG 6.1–7.8mmol/L, 2hPG 7.8–10 mmo/L). After infection control, oral hypoglycemic agents or insulin are selected according to blood glucose levels. Subjects are given second-generation cephalosporins empirically for anti-infection, while those who were allergic to cephalosporins are given Levofloxacin and then given sensitive antibiotics based on the results of drug sensitivity. Antibiotics are used for 7 to 14 days depending on the patient's condition.

#### 3.1.1 Experimental group

Subjects in the experimental group take modified BXXXD granules (10 g) orally twice daily for 2 weeks, after breakfast and supper.

#### 3.1.2 Control group

Subjects in the control group are given placebo granules (10 g) twice daily for 2 weeks.

#### 3.1.3 Concomitant medications

Subjects are not permitted to take other CHM during the entire study.

### 3.2 Collection of blood for metabonomics

The subject blood samples are collected in a centrifuge tube and placed at 37°C for 1 hour for coagulation and stratification. The supernatant is centrifuged and transferred to a freezing tube, and stored at –80°C for preservation. All samples are destructed after use.

### 3.3 Outcome measures

#### 3.3.1 Primary outcome

Disease outcomes include cure, significant effect, effective, and ineffective. The main result is the total clinical effectiveness after treatment (day 14) of the 2 groups, that is, the sum of cure rate, apparent efficiency and effective.

#### 3.3.2 Secondary outcomes

1) **TCM symptom score scale (**at days 4, 8, and 14).
2) **Pneumonia severity index** (at days 4, 8, and 14).
3) **usage time of antibiotic** (at days 6, 10, and 14).
4) **time required for blood sugar to reach the required level (**at days 2, 4, 6, 8, 10, 12, 14).
5) **frequency of hypoglycemia (**at days 2, 4, 6, 8, 10, 12, 14).
6) **laboratory examination:** including white blood cell count, percentage of neutrophils, C-reactive protein and procalcitonin **(**at days 4, 8, 14).
7) **chest CT (**at days 14).
8) ** Exploratory outcome:** Differences in blood metabolomics between the 2 groups after treatment **(**at days 14).

### 3.4 Safety assessment

The dose of modified BXXXD used in this study is within the recommended range based on the People's Republic of China Pharmacopeia (2015 edition). Furthermore, we take a variety of measures, including laboratory tests and subjective descriptions to evaluate the presence of gastrointestinal intolerance and liver or kidney dysfunction.

### 3.5 Compliance

Once subjects are randomized, investigators will try to follow them throughout the study. At each visit, adherence to intervention are monitored and subjects are required to return all unused packs of granules. Furthermore, subjects will be served with continuous support in subsequent stages, such as post-discharge rehabilitation advice.

### 3.6 Adverse events

Adverse events are defined as unanticipated or negative clinical features following treatment. Subjects are required to inform investigators of any abnormal reactions during the trial. In case of any severe adverse events, the intervention will be stopped immediately and a detailed description of severity, time, relationship with the drug, and the measures adopted derived from standard operational procedures of the China Food and Drug Administration will be recorded detaily in CRFs.

### 3.7 Data management and quality control

The researches in the study team have been asked for attending a training seminar before recruitment. Each one obtains a copy of the study protocol and they are required to comply with the protocol throughout the study period.

All data are recorded and gathered on CRFs. Any corrections or changes to the data written in CRFs should be documented and dated. All records are entered into a password-protected and pre-designed database by administrators who are blind to the group assignment. Two data administrators input and proofread the data independently to ensure the accuracy of the data. In addition, the Sichuan TCM evidence-based medicine center (Chengdu, China), which has no competitive interest, will be in charge of monitoring the data. The Department of Science Research of Affiliated Hospital of Chengdu University of TCM, which is independent of the research team, will perform data audits during the trial.

### 3.8 Statistical analysis

Statistical analysis will be performed at the Sichuan evidence-based Medicine Center of TCM using Statistical Package for the Social Sciences version 22.0 (SPSS 22.0, Chicago, IL). Intention-to-treat and per protocol sets are applied to analyse the efficacy of modified BXXXD. A safety analysis set is used to assess the safety of the study. Missing values are estimated using the principle of the last observation carried forward, with the data from the last study follow-up used as the final results.

Categorical data are tabulated with frequencies or percentages, and continuous data are reported as median or mean ± standard deviation. For the sociodemographic data and baseline variables are conducted using analysis of variance and Chi-squared test. To compare the variables of the same group before and after treatment, a paired *t*test is used. Repeated measures analysis of variance is used to compare the differences among the 2 groups. All statistical tests are bilateral tests and *P* values < .05 is regarded as statistical significance.

## 4 Discussion

In China, T2DM shows the “4 high” characteristics of high morbidity, high disability, high mortality and high disease burden. What more, with the increase in the prevalence of T2DM, the prevalence of CAP has also increased significantly.^[8–9]^ Although insulin and antibiotics have a good effect on the treatment of the disease in theory, these measures still cannot completely control the progress of the disease in practice, mainly due to insulin resistance and/or antibiotic resistance. CHM has been widely used in the treatment of diabetes or pulmonary infection in China for a long time due to its low probability of adverse reactions. Our research group has been engaged in TCM treatment of diabetes and related complications nearly 20 years. BXXXD has been proved to be effective in clinic and experimental studies.^[28–30]^ Modified BXXXD is suitable for pulmonary infection in patients with diabetes according to the theory of TCM. It has the effects of regulating lung qi, resolving phlegm, relieving cough and relieving asthma. However, the efficacy and safety of modified BXXXD in the treatment of T2DM with CAP have not been universally recognized. Thus, a prospective trial is needed to conclusively determine the effectiveness and safety of modified BXXXD for T2DM-CAP.

To the best of our knowledge, this is the first clinical study to investigate the efficacy of modified BXXXD combined with western medicine in the treatment of T2DM-CAP. In our study, we will employ validated objective tools such as TCM symptom score scale and pneumonia severity index. These measurements improve the reliability and generality of the results. In addition, this trial will also probe into the pathogenesis of T2DM-CAP and the action mechanism of TMC from the perspective of metabonomics. Although the study has limitations such as small sample size, short treatment period, and single-center design. The results of the trial will provide a preliminary objective evidence for the efficacy of modified BXXXD of T2DM-CAP. In the future, a multicenter randomized controlled trial with a large sample and the implementation of multidimensional comprehensive evaluations should be performed.

## Author contributions

**Conceptualization:** Hongjing Yang, Zhu Zeng.

**Investigation:** Xiaoying Huang, Ning Ding, Xiaoqin Long.

**Supervision:** Rensong Yue.

**Writing – original draft:** Hongjing Yang, Jie Zhou.

**Writing – review & editing:** Rensong Yue, Lizhen Wang.
